# Implementation and evaluation of short peripheral intravenous catheter flushing guidelines: a stepped wedge cluster randomised trial

**DOI:** 10.1186/s12916-020-01728-1

**Published:** 2020-09-30

**Authors:** Samantha Keogh, Caroline Shelverton, Julie Flynn, Gabor Mihala, Saira Mathew, Karen M. Davies, Nicole Marsh, Claire M. Rickard

**Affiliations:** 1 School of Nursing and Centre for Healthcare Transformation, Queensland University of Technology, Brisbane, QLD Australia; 2 Alliance for Vascular Access Teaching and Research (AVATAR), Menzies Health Institute Queensland, Griffith University, Brisbane, QLD Australia; 3 Nursing and Midwifery Research Centre, Royal Brisbane and Women’s Hospital, Brisbane, QLD Australia; 4 Centre for Applied Health Economics, Menzies Health Institute Queensland, Griffith University, Brisbane, QLD Australia; 5 School of Medicine, Griffith University, Brisbane, QLD Australia; 6 School of Pharmacy, University of Queensland, Brisbane, QLD Australia; 7 School of Nursing and Midwifery, Griffith University, Brisbane, QLD Australia

**Keywords:** Catheter-related infection, Evidence-based practice, Flushing, Peripheral intravenous catheter, Randomised trial

## Abstract

**Background:**

Peripheral intravenous catheters (PIVCs) are ubiquitous medical devices, crucial to providing essential fluids and drugs. However, post-insertion PIVC failure occurs frequently, likely due to inconsistent maintenance practice such as flushing. The aim of this implementation study was to evaluate the impact a multifaceted intervention centred on short PIVC maintenance had on patient outcomes.

**Methods:**

This single-centre, incomplete, stepped wedge, cluster randomised trial with an implementation period was undertaken at a quaternary hospital in Queensland, Australia. Eligible patients were from general medical and surgical wards, aged ≥ 18 years, and requiring a PIVC for > 24 h. Wards were the unit of randomisation and allocation was concealed until the time of crossover to the implementation phase. Patients, clinicians, and researchers were not masked but infections were adjudicated by a physician masked to allocation. Practice during the control period was standard care (variable practice with manually prepared flushes of 0.9% sodium chloride). The intervention group received education reinforcing practice guidelines (including administration with manufacturer-prepared pre-filled flush syringes). The primary outcome was all-cause PIVC failure (as a composite of occlusion, infiltration, dislodgement, phlebitis, and primary bloodstream or local infection). Analysis was by intention-to-treat.

**Results:**

Between July 2016 and February 2017, 619 patients from 9 clusters (wards) were enrolled (control *n* = 306, intervention *n* = 313), with 617 patients comprising the intention-to-treat population. PIVC failure was 91 (30%) in the control and 69 (22%) in the intervention group (risk difference − 8%, 95% CI − 14 to − 1, *p* = 0.032). Total costs were lower in the intervention group. No serious adverse events related to study intervention occurred.

**Conclusions:**

This study demonstrated the effectiveness of post-insertion PIVC flushing according to recommended guidelines. Evidence-based education, surveillance and products for post-insertion PIVC management are vital to improve patient outcomes.

**Trial registration:**

Trial submitted for registration on 25 January 2016. Approved and retrospectively registered on 4 August 2016. Ref: ACTRN12616001035415.

## Background

Vascular access via peripheral intravenous catheters (PIVCs) in the hand or arm is frequently required during hospital care to administer hydration fluids, medication, and blood transfusions. An estimated 70% of hospitalised patients will require a PIVC [1], and a large portion of these will be inserted and cared for by nurses [2]. Historically, vascular access device research and practice has focused on reducing bloodstream infection rates, particularly in central venous catheters [3]. However, catheter-related bloodstream infection rates in PIVCs are extremely low, at 0.03–0.1% [4, 5], or 0.5 per 1000 catheter days [4], whereas PIVC failure rates due to occlusion, infiltration, dislodgement, phlebitis, or infection are 36% [6].

A failed PIVC is distressing for patients who have to endure the discomfort of a PIVC complication as well as the pain associated with a PIVC replacement. Complications and failure also hold implications for healthcare budgets with an estimated per-patient cost for PIVC replacement of AUD $70 (~ US $50) in 2012 [7], and additional costs associated with an extended hospital stay due to delayed or interrupted intravenous therapy. With over two billion PIVCs purchased globally every year [8], and based on current PIVC complication rates, millions of dollars are wasted each year through PIVC failure. A reduction of PIVC failure by just 10% could save significant healthcare dollars and reduce unnecessary discomfort and risks for patients.

Standards of practice for PIVC maintenance globally include statements pertaining to flushing for maintaining patency and function [9, 10]. Flushing the catheter before and after administration of intravenous medication creates opportunity for assessment of insertion site and catheter performance and helps maintain catheter patency through avoiding mixing of incompatible fluids and medications, as well as minimising build-up of fibrin and biofilm that contribute to thrombophlebitis and catheter dysfunction [9, 11]. However, practice surveys and observational studies have revealed significant variation in practice (e.g. use of syringes smaller than 10 mL, use of heparinised saline) and other variations from guideline recommendations. These inconsistencies and failure to implement recommended practices likely contribute to current relatively high PIVC failure rates [12–14]. Consequently, we developed a multifaceted intervention to raise awareness of the risks associated with PIVC use and adherence to guidelines for PIVC flushing practice. The aim of this study was to evaluate the effect of a multifaceted intervention that ‘bundled’ reinforcement of PIVC flushing guidelines with manufacturer-prepared pre-filled flush syringes.

## Methods

### Study design

This study was a single-centre, sequential, incomplete, stepped wedge, cross-sectional cluster randomised trial (SWCRT) with an implementation phase. Clusters (wards) were randomised for both the control and the intervention phases. Wards had equal exposure periods in each phase, rather than for the duration of the study (incomplete), which helped to avoid the potentially confounding impact of different levels of exposure to the intervention seen in the complete SWCRT design, as well as minimising the measurement burden [15]. As is characteristic of the SWCRT designs, each cluster experienced both control and intervention condition, but with different participants observed in each phase (cross-sectional). No data was collected during the implementation (transition) phase when staff received education about guidelines and use of pre-filled syringes. The SWCRT design was chosen over a traditional randomised controlled design due to an inability to minimise contamination between groups if randomising at a patient level. Further, the SWCRT design was preferred over the classic parallel-cluster design due to optimised feasibility and management of staggered rather than en bloc roll out of intervention [16].

The trial was undertaken at a single metropolitan hospital in Queensland, Australia: the Royal Brisbane and Women’s Hospital (RBWH) is a 929-bed quaternary and tertiary referral teaching hospital and the largest provider of health care services for Queensland Health. The trial received ethics approval from the Griffith University (GU Ref No: 2016/052) and the Royal Brisbane and Women’s Hospital (HREC/15/QRBWH/592) Human Research Ethics Committees. This trial was registered with the Australian New Zealand Clinical Trials Registry, number ACTRN12616001035415.

### Participants

Research nurses (ReNs) screened medical and surgical departments for participants. Patients were eligible if they were 18 years or older and required a PIVC for clinical treatment for longer than 24 h (with or without continuous infusion). Non-English-speaking patients without interpreters and patients who had an existing PIVC-associated infection were excluded.

Given the intervention was a reiteration of current practice recommendations and included already licenced products, no new practice was introduced and no treatment was being withheld; the ethics committees granted a waiver of consent due to the low/negligible risk associated with the trial. Patients were given information sheets about the study being conducted on the ward and had the ability to opt out if they wished.

### Randomisation and masking

Cluster randomisation was performed once at the commencement of the trial using a computer-generated sequence (Microsoft Excel), with allocation concealed (from wards) until each approached the time of crossover to the implementation phase. Blinding of patients and clinical and research nurses was not possible for this intervention. However, infection outcome was undertaken by blinded laboratory scientists and infectious disease specialists.

### Procedures

**Fig. 1:**
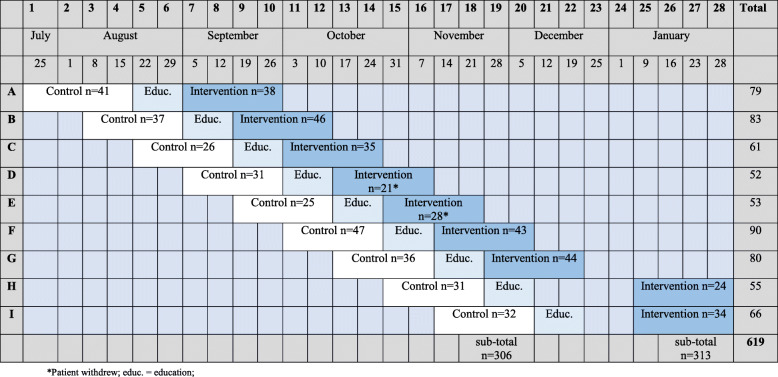
Incomplete stepped wedge cluster randomised trial with an implementation period. Study design and patient flow

ReNs screened potential patients for eligibility using a screening tool reflecting the inclusion and exclusion criteria. Recruitment and data collection for the control period continued until 35 patients were recruited in each group on each ward. Following the implementation phase for that ward, data was collected for 4 weeks in the intervention period until a sample of 35 was obtained. This occurred in a staggered and sequential pattern as per randomisation schedule and schema (Fig. 1).

As per the SWCRT design, the study wards were allocated to both the control and intervention periods. The control group was the current ‘as usual’ (variable) care. Practice in the control arm included a combination of manually prepared syringes with 0.9% sodium chloride of varying volumes, or infusions from 100 mL or 1000 mL 0.9% sodium chloride bags as per clinician preference and in variable frequency.

The intervention group received maintenance (flushing) practice according to PIVC flushing guideline recommendations based on Infusion Nurses Society (INS) Standards of practice [9] and included manufacturer-prepared pre-filled flush syringes. The intervention (Table 1) was centred on raising awareness of the importance of PIVC flushing, reinforcing, and supporting adherence to current practice recommendations as follows: assessment of PIVC catheter and site; flushing pre- and post-medication administration, and also 8 hourly (as per site-specific guidelines) if the PIVC was not being used (for infusion or regular medications): use of 0.9% sodium chloride solution in a single dose manufacturer-prepared pre-filled syringe (with properties of a 10 mL syringe to minimise applied pressure); using a volume equal to twice the internal volume of the catheter system; delivered using a gentle, pulsatile technique; and documentation. The INS recommendation for initial aspiration of blood is for central vascular access devices and is not recommended for PIVCs.

**Table 1:** Summary of the trial intervention

Education delivered via (i) verbal presentation with slide deck, (ii) practical demonstration, and (iii) electronic resources. Guidelines for PIVC flushing • Assessment of catheter and site • Pre/post drug administration • At least Q8h if not in use* • Use of single-dose manufacturer-prepared prefilled syringe^†^ • Volume of at least X2 the length and diameter of the catheter^‡^ • Use of gentle, pulsatile technique • Documentation of flush (medication or fluid chart)	

*As per study site policy

^†^With properties of 10 mL syringe

^‡^Except post blood transfusion and some drugs

The educational component for the implementation period was delivered via three sessions a week over a 2-week period during shift handover (morning/afternoon) to ensure minimum 80% nursing staff attendance. The educational component reiterated the current policy and practice recommendations related to PIVC flushing and comprised of the following:

(i) Presentation: written guidelines were summarised for staff in a slide deck at an in-service delivered on the ward in person and in hard copy. (Reference: Royal Brisbane and Women’s Hospital 05450/ Proc: Peripheral Intravenous Cannulation, Venepuncture and Infusions- Adult and Paediatrics Version 6 Effective date: 12/2015 Review date: 12/2018.)
(ii) Industry education and demonstration: this entailed a representative from Becton Dickinson (BD Medical) educating and training staff on the use of manufacturer-prepared pre-filled flush syringes used in the study (Posiflush™, BD Medical, Sandy UT, USA).
(iii) Electronic information: copies of the information related to the study, as well as slideshow presentation, and the intervention (guidelines and pre-filled syringe use) were distributed via a group email from each ward’s nurse unit manager. Links to external films were not possible due to firewalls of the institutional computer networks.

PIVCs were inserted by ward nurses and doctors without the use of ultrasound according to standard operating procedures. PIVC site, catheter gauge, and attachments were chosen by the inserter depending on the needs of each individual patient. Pre-insertion skin disinfection was done using SoluPrep Swabs (2% chlorhexidine in 70% isopropyl alcohol; 3 M St Paul, MN, USA). PIVCs were short (25 to 29.5 mm) non-winged Insyte Autoguard BC with Blood Control Technology (BD Medical). SmartSite needle-free valves (BD Medical) were connected to PIVCs directly or via a 10-cm extension set with bonded 3-way Connecta (BD Medical).

One PIVC was assessed per patient (the first PIVC inserted after the patient met the inclusion criteria). All post-insertion care was provided by clinical staff. ReNs recorded patient and device outcomes and advised staff about study interventions and products before the study and during the trial. The decision to remove PIVCs was made by clinical staff. PIVCs were removed on completion of therapy, when a complication occurred, or routinely at 72–96 h of dwell time. Local policy allowed for dwell time to be extended to more than 96 h if the PIVC was still required, there were no signs or symptoms of catheter dysfunction or infection were observed, and the decision was clinically justified.

ReNs visited patients daily while the PIVC was in place and followed up on any observed or reported adverse events. Baseline patient and PIVC characteristics were recorded. During PIVC dwell, data were collected on PIVC therapy, insertion site condition, and PIVC performance, e.g. patency. At PIVC removal, ReNs recorded complications, dwell time, and clinical variables. Forty-eight hours after PIVC removal, ReNs checked the hospital pathology system for blood, PIVC tip, or insertion site culture results.

All observations were entered into a secure, portable electronic device using the REDCap electronic data capture tools hosted at Griffith University. REDCap (Research Electronic Data Capture) is a secure, web-based software platform designed to support data capture for research studies [17]. Clinical staff did not have access to REDCap. A project manager audited data quality, completeness, and protocol adherence.

As per routine clinical practice, medical staff ordered blood, PIVC tip, or site swab cultures if patients were suspected to have PIVC-associated infections. These samples were obtained by bedside nurses, processed in the hospital microbiological laboratory by blinded staff, and results were accessed by researchers.

### Outcomes

The primary outcome was all-cause PIVC failure as a composite of any unplanned PIVC removal, prior to completion of therapy. This included occlusion (PIVC would not infuse, or leakage occurred around the site when fluid is injected), infiltration (leaking of fluid into surrounding tissues), dislodgement (complete), phlebitis (defined by the presence of two or more of the following symptoms: pain, redness, swelling, and a palpable cord), and infection (local or laboratory-confirmed bloodstream infection [3]). A composite measure was chosen as it increases precision and efficiency, as PIVC failure is the outcome of importance to patients and reduced patency taking various pathways to the same endpoint [18].

Secondary outcomes were subtypes of PIVC failure (occlusion, infiltration, dislodgement, phlebitis, and infection), PIVC dwell time (time from insertion to removal), and cost per patient (direct cost to the hospital for device management, cost of PIVC replacements and related adverse events). Adverse and serious adverse events (i.e. death, admission to intensive care, or laboratory-confirmed bloodstream infection) were monitored using hospital records and reported to the human research ethics at the study site.

Cost analysis included labour and material costs used for initial PIVC insertion and staff time taken for this, cost of flush or infusion products, and additional cost of follow-on PIVC insertions due to PIVC failure. Details of the cost analysis are shown in Additional file: Table S1. Purchase and other costs were collected at 2016 Queensland Health prices in Australian dollars. Mean total costs and timing for control and intervention products were based on data from an observational study of infusion and flushing methods we had conducted just prior to commencement of this trial [14], and also a comparative study [19]. Costs for nursing and medical staff time were based on published staff salaries (Queensland Health 2016) [7]. Staff satisfaction was collected via a study-specific survey on product use as well as utility and impact of the educational intervention. The survey comprised six items with an 11-point (minimum 0 to maximum 10) scale and one open-ended question for free-form text responses.

### Statistical analysis

Recent trials showed PIVC failure at the study institution were approximately 40% using current flushing practices [5, 20]. An absolute 10% reduction in the proportion of PIVC failure to 30% was considered both clinically important and feasible. For a two-sample comparison with 90% power, two-sided α level of 0.05, a sample of 35 patients in each group from 9 wards (35 each ward each group) was required (using the Stata command ‘steppedwedge’ [21]). The calculation assumed an intra-cluster correlation coefficient of 0.05 and coefficient of variation (of clusters) of 0.27 [15]. Thus, the total sample required was 630.

Data cleaning involved checks of missing, outlier, and improbable values. Quantitative data was exported into Stata 15 (Stata-Corp, Texas). Descriptive statistics were summarised to determine equality at baseline between study groups. Categorical data were summarised as counts and proportions, and continuous data as means and standard deviations. Incidence rates per 1000 catheter days, rate ratio, and log-rank test were presented by study groups. Univariable Cox regressions were used to identify potential confounders at the *p* < 0.20 level. At this step, the effect of individual wards and their sequence were also tested. The potential confounders were carried forward to the multivariable analysis. Various analytical approaches were tested, including study group and cluster treated as fixed effects, interaction between groups and clusters, and modelling with unobservable cluster-level random effect. Cox regression with study group as fixed effect was chosen. Standard errors were estimated allowing for intragroup correlation (i.e. observations were independent across clusters but not necessarily within clusters). Potential confounders were dropped from the multivariable model, in a manual one-by-one stepwise manner, at *p* ≥ 0.05. The specification of the final model was link-tested and the proportional hazard assumption was tested with the Schoenfeld residuals and the log-log plot of survival. Secondary outcomes were compared using hypothesis tests. Intention-to-treat principle was followed. Missing values were not imputed. Two-tailed *p* values of < 0.05 were considered as statistically significant.

A cost analysis was done from the perspective of public hospitals, as they are the leading providers of health care in Australia and main purchasers of PIVCs and products used for ongoing maintenance. Total resource use and costs were calculated for each group. The mean cost of PIVCs was calculated using the Zhao and Tian host command in Stata [22]. This estimator is appropriate for estimating mean costs that allow adjustment for censored data. Start times for costs was the date and time of PIVC insertion. Dwell time was defined as the interval from the start time until the date and time of PIVC removal. Costs of insertion were assumed to be equal between groups as not impacted by intervention as costed accordingly. The dwell time was censored for determining the total maintenance and replacement costs. The incremental net benefit was calculated using two-stage nonparametric bootstrap sampling for clustered data [23]. Survey data was summarised using counts and percentages with a simple thematic analysis of textual data.

### Role of the funding source

The funder of the study and education provider had no role in the study design, data collection, data analysis, or writing of the manuscript. The corresponding author has full access to all the data in the study and had final responsibility to submit for publication.

## Results

Between July 2016 and February 2017, we screened 630 patients, and 619 eligible patients were consecutively enrolled. Reasons for non-participation included the following: did not meet inclusion criteria (*n* = 5), declined (*n* = 4), and no reason stated (*n* = 2). Nine wards participated in the study (306 patients in the control group and 313 in the intervention group). Computerised randomisation determined the order of sequential rollout. Allocation was concealed until each ward was in the implementation period. Figure 1 details the list and sequence of clusters, as well as the number of participants by period. Two patients from the intervention group withdrew, and thus, a total of 617 patients were analysed. Study ceased when recruitment complete.

A total of 38,712 PIVC dwell hours (1613 days) were studied, with a mean dwell time 2.6 days (standard deviation [SD] 1.6) which was similar in the study groups. Clinical and demographic characteristics were also similar between groups (Table 2).

**Table 2:** Baseline demographic and clinical characteristics

	Control (***n*** = 306)	Intervention (***n*** = 313)	Total (***n*** = 619)
Age, years^a^	60 (19)	58 (19)	59 (19)
Sex: males	166 (54)	163 (52)	329 (53)
Diagnosis:			
Surgical	164 (54)	168 (54)	332 (54)
Medical	139 (45)	137 (44)	276 (45)
Other	3 (1)	8 (3)	11 (2)
Weight category:			
Under	55 (18)	61 (19)	116 (19)
Healthy	151 (49)	160 (51)	311 (50)
Over	87 (28)	79 (25)	166 (27)
Obese	13 (4)	13 (4)	26 (4)
Three of more co morbidities:	164 (54)	171 (56)	335 (54)
Abs. leucocytes < 1000 μL within 72 h	11 (4)	15 (6)	26 (5)
Infection at recruitment	24 (8)	31 (10)	55 (9)
Wound	99 (32)	94 (30)	193 (31)
Good skin integrity	107 (35)	130 (42)	237 (38)
Vein quality:			
Good	53 (43)	62 (47)	115 (45)
Poor	69 (57)	71 (53)	140 (55)
Antibiotic therapy at recruitment	153 (50)	125 (40)	278 (45)
IV therapy at recruitment:			
Fluids	147 (48)	162 (52)	309 (50)
Intermittent	38 (12)	26 (8)	64 (10)
None	121 (40)	125 (40)	246 (40)
Inserted on dominant side	159 (54)	148 (48)	307 (51)
Inserted by:			
Doctor	125 (65)	156 (73)	281 (69)
Nurse	58 (30)	48 (23)	106 (26)
Other	10 (5)	9 (4)	19 (5)
Device location:			
Hand	93 (30)	113 (36)	206 (33)
Cubital fossa	80 (26)	68 (22)	148 (24)
Posterior forearm	63 (21)	53 (17)	116 (19)
Wrist	38 (12)	50 (16)	88 (14)
Anterior forearm	30 (10)	25 (8)	55 (9)
Other	2 (1)	4 (1)	6 (1)
Multiple insertion attempts	42 (26)	45 (24)	87 (25)
Skin preparation:			
Chlorhexidine 2% with 70% alcohol	118 (89)	149 (96)	267 (93)
Other	14 (5)	6 (2)	20 (3)
Device size (gauge):			
22	95 (31)	105 (34)	200 (32)
20	163 (53)	168 (54)	331 (54)
18	47 (15)	40 (13)	87 (14)
16	53 (43)	62 (47)	115 (45)
Extension tubing	216 (71)	205 (65)	421 (68)
Three-way tap	220 (72)	212 (68)	432 (70)

Frequencies and column percentages shown, unless otherwise noted

*abs* absolute, *μL* microlitre

^a^Mean (standard deviation)

Ninety-one (30%) patients in the control group experienced PIVC failure compared to 69 (22%) patients in the intervention group (risk difference: − 8%, 95% CI − 14 to − 1, *p* = 0.032). The difference between PIVC failure per 1000 PIVC days was not significant (control 110 [95% CI 89.4 to 135], intervention 87.9 [95% CI 69.4 to 111], incidence rate ratio 0.80 [95% CI 0.58 to 1.11], *p* = 0.192) (Table 2).

Other than leakage, no significant differences in the incidence of occlusion, infiltration, dislodgement, or phlebitis were identified between groups (Table 2). No PIVC-related bloodstream infections were reported in either group.

The risk of PIVC failure was significantly lower (hazard ratio [HR] 0.78 [95% CI 0.63 to 0.97], *p* = 0.029) in the intervention group. Additionally, the use of extension tubing was associated with a reduction in failure (HR 0.71 [95% CI 0.57 to 0.87], *p* = 0.001). Any infection at baseline was associated with an increased risk of PIVC failure (HR 1.68 [95% CI 1.1 to 2.58], *p* = 0.017). The impact of ward sequence on outcome was neutral (HR 1.01 (95% CI 0.88 to 1.14), *p* = 0.93) (Table 3).

**Table 3:** Study outcomes by treatment group

	Control, ***n*** = 306	Intervention, ***n*** = 311	***p*** value
PIVC failure	91 (30)	69 (22)	*0.03*
Device-time (accumulative days)	828	785	0.25
Incidence rate per 1000 device days	110 (89.4–135)	87·9 (69.4–111)	
Incidence rate ratio	Referent	0.80 (0.58–1.11)	0.19
Secondary outcomes:			
Occlusion	17 (6)	18 (6)	0.90
Leakage	17 (6)	7 (2)	*0.03*
Infiltration	40 (13)	32 (10)	0.28
Dislodgement	22 (7)	19 (6)	0.59
Phlebitis (research definition)	20 (9)	11 (5)	0.11
Death	3 (1)	1 (0)	0.30
Positive blood culture	4 (1)	4 (1)	0.98
Cox regression analysis			
Covariate	**Univariable Cox regression**^a^ *n* = 617	**Multivariable Cox regression**^b^ *n* = 617	
Study group: intervention	0.81 (0.59–1.11)*	0.78 (0.63–0.97)^†^	
Ward sequence (increase by one)	1.01 (0.88–1.14)	^$^	
Infection at baseline	1.68 (1.06–2.66)^†^	1.68 (1.10–2.58)^†^	
Extension tubing	0.72 (0.52–1.00)*	0.71 (0.57–0.87)^†^	

Frequencies and column percentages shown, unless otherwise noted; *p* values calculated using chi-squared, rank-sum, or log-rank tests

*p < 0.20

^†^*p* < 0.05

^a^No other adjustments other than covariate listed

^b^Adjusted for within-cluster correlation

Eight (3%) of 306 patients in the control group and six (2%) of the 311 patients in the intervention group had serious adverse events (death [*n* = 4], intensive care admission [*n* = 1], or infection [*n* = 8]). One patient had fever of unknown origin with no aetiology recorded. None of these outcomes was considered to be associated with study participation.

Cost analysis results are presented in Table 4. The mean product cost for control group PIVCs was AUD $13.42 and a staff (time) cost of $7.47. The average costs for the products and staff were $1.98 and $7.07, respectively, for the intervention PIVCs. The replacement cost of PIVC was $13 which had been calculated in a previous study [7]. The overall cost was lower in intervention group $22.33 (95% CI 22.18 to 22.49, *p* < 0.001) compared to the control group $33.39 (95% CI 33.28 to 33.50, *p* < 0.001), with a statistically significant difference in the total cost between groups of $11.05 (95% CI 10.86 to 11.24, *p* < 0.001) per patient. The incremental net benefit calculated was $12.02 (95% CI 11.04 to 12.99).

**Table 4:** Mean costs per patient and cost-effectiveness associated with PIVC maintenance

	Control	Intervention
Product costs^a^	$13.42	$1.98
Staff time^a^	$7.07	$7.47
Mean maintenance costs	$29.60 (28.88–30.32) *p < 0.001*	$14.84 (14.38–15.31) *p < 0.001*
Difference in mean costs between intervention and control		$14.75 (13.90–15.61) *p < 0.001*
Cost of replacement PIVC^b^	$13	$13
Mean maintenance and replacement costs^c^	$33.39 (33.28–33.50) *p < 0.001*	$22.33 (22.18–22.49) *p < 0.001*
Difference in mean costs between intervention and control	–	$11.05 (10.86–11.24) *p < 0.001*

*PIVC* peripheral intravenous catheter

^a^Standard deviations were not reported as weighted mean times were calculated

^b^Replacement PIVCs were required by 91 patients in the control group and 69 patients in the intervention group

^c^Mean cost estimated using hcost program for censored data: Australian Dollar values shown (2016)

**Fig. 2:**
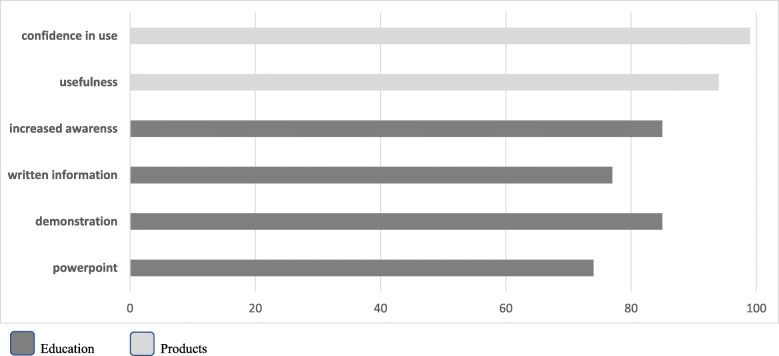
Staff satisfaction rating of intervention % scored ≥ of 7 (out 10)

Ward nurse satisfaction scores were generally high for both the educational intervention and the use of manufacturer-prepared pre-filled flush syringes (see Fig. 2). The majority of nurse respondents indicated that they found the educational intervention useful in all its forms (slideshow presentation 48/65, 74% rates ≥ 7; demonstration 68/80, 85% rated ≥ 7; and written information 64/83 77% rated ≥ 7). The majority also stated that it raised their awareness about flushing guidelines and policy (62/83, 85% rated ≥ 7). The utility and confidence in the use of manufacturer-prepared pre-filled flush syringes in practice was also rated highly (80/85, 94% rated ≥ 7 usefulness and 84/85, 99% rated ≥ 7 confidence in use). Results are graphically displayed in Fig. 2. The main themes emerging from textual responses to an open question inviting (any) further comment were the ‘time saving’ and ‘convenience’ factor of the manufacturer-prepared pre-filled flush syringes.

## Discussion

This study demonstrated that a multifaceted intervention combining clinical education, reinforcement of PIVC flushing guidelines, and complementary product use with manufacturer-prepared pre-filled flush syringes reduced both the proportion and risk of PIVC failure. The concept of *bundling* key steps from lengthy practice guidelines into a set of point-of-care reminders to improve staff compliance is not new. Following the reported success of central venous access device (CVAD) bundles [24], a range of PIVC insertion and maintenance bundles have also been developed and implemented into practice, in pursuit of similar clinical improvements. Many studies reported significant reductions in PIVC-related bloodstream infection, while other studies reported reduced incidence of other PIVC-related complications (e.g. phlebitis, infiltration) [25]. However, none of these studies used a randomised design, and standardisation was less evident in PIVC studies compared to CVAD studies. Nevertheless, there is evidence that a combination approach to interventions or the processes of care delivery is an advantageous approach that consistently yields improved outcomes compared to single-entity interventions [25, 26].

Some single-entity intervention studies have reported improved outcomes, but there are caveats related to reporting and study design. A systematic review comparing the effectiveness of pre-filled to manually prepared flushes showed a reduced rate of catheter-related bloodstream infection (CRBSI) and occlusions, as well as extending PIVC dwell with the pre-filled group [27]. However, the authors stated that these results should be treated with caution due to poor quality reporting and a number of limitations with the reviewed studies (e.g. unit of measurement, lack of core outcome definitions, heterogeneity). A recent pre- and post-test study observed a decrease in PIVC failure associated with the use of pre-filled syringes [28]. However, the overall failure rate even post-intervention remained high (43%) indicating that there are other drivers at play related to PIVC failure. In the randomised trial reported in this manuscript, manufacturer-prepared pre-filled syringes were part of a *whole-of-practice* approach to PIVC flushing. The results of the trial reported in this publication showed that even with relatively lower complication rates pre-intervention (30%), streamlining practice and products related to PIVC flushing yielded significantly reduced proportion and risk of failure. We demonstrated that raising awareness about the importance of the maintenance of vascular access and the techniques to do this, coupled with products that facilitate adherence with practice recommendations minimised variation in practice.

Nursing staff rated the educational intervention and the use of pre-filled flush syringes highly. The convenience and time-saving element were frequently cited. This aligns with previous comparison research [19]. Other possible benefits from the use of manufacturer-prepared pre-filled flushes that potentially augmented the impact of the educational intervention likely relate to reduced risk of device or solution contamination due to negated need to draw up solution. Additionally, pre-filled syringes offer improved medication safety due to pre-labelling, reduced intra-device reflux due to positive pressure plunger, reduced risk of cannula or vessel injury (as the syringe has 10 mL properties with appropriate pressure as reflected by pounds per square inch regardless of volume/syringe size), reduced waste, and time savings.

Costs were significantly lower in the intervention group, and staff rated the educational intervention and use of manufacturer pre-filled flush syringes highly. The cost analysis for the control group was possibly inflated by the use of 100 mL bag infusions (as an alternative to flush delivery). However, this is not unique to this study setting. The staff time costs were higher in the intervention group when compared to standard practice that did not employ consistent flushing practice. It is likely that any small absolute cost difference between manually and manufacturer-prepared flushes when consistently employed (as per guidelines) is offset by a reduction in staff preparation time and lower PIVC failure rate [19]. Given the results of the study, many of the estimated 10 million PIVC failures and $175 million in re-siting costs attributed to these PIVC complications in Australia could be reduced. Transposing these figures globally where billions of catheters are used each year [2] underscores the broader and significant potential for improvement in clinical and cost-effectiveness associated with improved maintenance practice.

Limitations of this study are related to the single site setting of the trial and the sampling from medical and surgical wards. Though this captured some diversity in patient population (e.g. orthopaedics, respiratory medicine), patients receiving cancer care, critical care, and children were not included. This has some implications for generalisability of findings. Further, the incomplete design did not allow for continued evaluation of outcomes. Multi-site implementation and evaluation over time would enhance generalisability and sustainability of the findings. However, the incomplete design improved the efficiency of the study and ensured equal exposure of all clusters to the intervention, minimising the complexity of data analysis. The stepped wedge design also allowed for a more manageable and gradual implementation of the study protocol and data collection.

## Conclusions

PIVC outcomes are of high importance considering the significant level of use and the patient morbidity and economic burden to hospitals associated with complications. Research within vascular access has focused on the reduction of infection; however, the scale and implications of PIVC failure have only recently been acknowledged. This trial is one of the few recently published studies addressing the maintenance phase of the PIVC [20, 29, 30]. It has provided important information on the effectiveness of post-insertion PIVC maintenance, specifically, optimising patency through flushing. The post-insertion phase accounts for approximately 90% of the episode of vascular access care and is predominantly in the nursing domain. Important reductions in morbidity and healthcare costs could be achieved if a comprehensive post-insertion intervention focused on ongoing assessment and maintenance could be introduced nationally or worldwide. The impact that the quality of PIVC maintenance care has on reducing complications and failure and optimising device duration is significant.

## Supplementary information

**Additional file 1: Table S1**. Costs associated with each study group: products and staff time and costs of responding to failure.

## Data Availability

The datasets generated and analysed for the published study are not publicly available due to restrictions related to sponsor agreement and ethical guidelines but are available from the corresponding author as outlined. Formal requests for data sharing are considered in line with the sponsor and ethical guidelines, and with due regard given to funder. Requests are via a standard proforma describing the nature of the proposed research and extent of data requirements. Data recipients are required to enter a formal data-sharing agreement with Sponsoring institution, which describes the conditions for release and requirements for data transfer, storage, archiving, publication, and intellectual property. Requests are reviewed by the trial investigative team in terms of scientific merit and ethical considerations, including patients’ consent. Data sharing is undertaken if proposed projects have a sound scientific or patients’ benefit rationale. Restrictions relating to sharing of individual participant data to preserve patients’ confidentiality and consent will be managed by aggregating and/or anonymising identifiable patients’ data. Additionally, all indirect identifiers that could lead to deductive disclosures will be removed in line with the ethics committee and study Sponsor’s data-sharing guidelines.

## Abbreviations

AUD: Australian dollar
CI: Confidence interval
CRBSI: Catheter-related bloodstream infection
CVAD: Central venous access device
HR: Hazard ratio
INS: Infusion Nurses’ Society
PIVC: Peripheral intravenous catheter
RBWH: Royal Brisbane and Women’s Hospital
REDCap: Research Electronic Data Capture
SD: Standard deviation
ReN: Research nurse
SWCRT: Stepped wedge cluster randomised trial
