# Supplementary material for: Implementation and evaluation of short peripheral intravenous catheter flushing guidelines: a stepped wedge cluster randomised trial
Source: BMC Med. 2020 Sep 30;18:252. doi: 10.1186/s12916-020-01728-1 (PMC7526260; doi:10.1186/s12916-020-01728-1)
Supplement: Supplementary file 1 — Additional file 1: Table S1. Costs associated with each study group: products and staff time and costs of responding to failure. [file 12916_2020_1728_MOESM1_ESM.docx]

**Additional file: Table S1: Costs associated with each study group: products and staff time and costs of responding to failure**

|  | **Product(s)** | **Cost per unit in AU$** | **Cost per method AU$** | **Assumptions/**  **percentage of patients** |
| --- | --- | --- | --- | --- |
| Manually prepared flush | BD Luer Lock 10mL syringe  SYRINGE,HYPO:INJ,ST,SU,L/LK,10ML | $0·09 | $0·21 | 34% of control sample  @ X6 flushes/day  **= $1·26/day** |
|  | Kabi 10 mL 0.9% Sodium Chloride ampoule | $0·10 |  |  |
|  | BD Precision Glide Drawing up needle  NEEDLE, DRAW UP: BLUNT,ST,SU,L/LK,18GX38MM | $0·02 |  |  |
| 100mL bag & giving set | Baxter 100mL 0.9% Sodium Chloride | $1·49 | $9·74 | 44% of control sample @ X3 sets/day  **= $29·22/day** |
|  | BBraun Standard Giving set for pump  (Most would use an infusion pump and line) | $8·25 |  |  |
| 1000mL bag with burette and pump giving set | Baxter 1000mL 0.9% Sodium Chloride | $1·12 | $13·11 | 22% cost post medication flush (manually prepared) only into burette X3/day **=0·63/day** |
|  | BBraun Standard Giving set for pump | $8·25 |  |  |
|  | (Burette 150mL)  ADMIN SET,IV: BURETTE,150ML,1XS/SITE,56CM | $3·74 |  |  |
| Manufacturer prepared prefilled flush | BD Posiflush^TM^ 5mL 0.9% Sodium Chloride | $0·33 | $0·33 | 100% of intervention sample @ X6 flushes/day  **= $1‚·98/day** |
| PIVC | BD Insyte^TM^ Autoguard^TM^ Blood Control (non-winged) | $1·99 |  | 100% of study patients |
| PIVC failure costs^6^ |  | $69·70 |  |  |
| Staff costs updated to 2016^6^ | Registered nurse | $0·6228 |  | Per minute |
|  | Junior medical staff | 0·8692 |  | Per minute |
|  | Senior medical staff | $1·2702 |  | Per minute |

AU$ = Australian dollars; PIVC = peripheral intravenous catheter

Calculations:

Costs of insertion equivalent across groups (as products and time the same).

Weighted average of Control product costs = $13·42

i.e. (1·26 x 0·43) + (29·22 x 0·44) + (0·63 x 0·22)

Average of Intervention product costs is $1·98

Weighted average of staff time in Control is $7·07

i.e. [(1·76 mins x 6 events) x 0·34] + [(1·26 mins x 3 events) x 0·44] + [(1·76 mins x 6 events) x 0·22]

Average intervention time cost is 1·25 mins x 6 events = $7·47

END
